# Retrospective Evaluation of Novel Synthetic Opioids and Xylazine Chronic Intake by Post‐Mortem Hair Testing

**DOI:** 10.1002/dta.3852

**Published:** 2025-01-21

**Authors:** Marco Ballotari, Nicola Pigaiani, Anna Bacci, Karen S. Scott, Gregory G. Davis, Rossella Gottardo, Federica Bortolotti

**Affiliations:** ^1^ Unit of Forensic Medicine, Department of Diagnostics and Public Health University of Verona Verona Italy; ^2^ Division of Forensics, Department of Pathology University of Alabama at Birmingham Birmingham Alabama USA; ^3^ Division of Laboratory Medicine, Department of Pathology University of Alabama at Birmingham Birmingham Alabama USA

**Keywords:** fentanyl, nitazenes, nonpharmaceutical fentanyls, opioid overdose, post‐mortem hair testing, xylazine

## Abstract

Fentanyl and its derivatives (nonpharmaceutical fentanyl, NPFs) represent the largest group among synthetic opioids. Fentanyl‐related deaths and fatalities from tampering with pharmaceutical products have been reported. Furthermore, in the United States, adulterants such as xylazine and other substances, including the nitazenes class of opioids, have been found in an increasing number of unintentional overdose deaths, drug seizures, and reports of use by recreational drug users. Monitoring the diffusion of fentanyl, NPFs, nitazenes, and adulterants among the population is a fundamental pursuit in forensic toxicology. The use of hair analysis is perfect for this purpose, providing essential information regarding previous intake or exposure to xenobiotics. The present study focused on the development and validation of a UPLC‐MS/MS method for the detection and quantification of fentanyl, NPFs, and xylazine, as well as the semiquantitative detection of nitazenes in hair samples from post‐mortem cases collected under the jurisdiction of the Jefferson County Coroner/Medical Examiner's Office (Birmingham, AL, USA). The method was validated according to international guidelines and applied to the analysis of *n* = 250 post‐mortem hair samples. In 52% of the analyzed hair samples, fentanyl, its main metabolites, and related analogs were detected, showing significant exposure to these substances in the population. Moreover, xylazine was detected in *n* = 48 hair samples (19.2%). The developed UPLC‐MS/MS method proved suitable for rapid chromatographic separation and sensitive detection of the studied compounds. In addition, this is the first time that xylazine and protonitazene have been measured in hair samples of subjects exposed to synthetic opioids.

## Graphical abstract

The study presents a validated UPLC‐MS/MS method applied for the detection of fentanyl, NPFs, xylazine, nitazenes in hair samples from 250 post‐mortem cases collected at the Jefferson County Coroner/Medical Examiner's Office (Birmingham, AL, USA). In 52% of the analyzed hair samples, fentanyl, its main metabolites, and related analogs were detected.

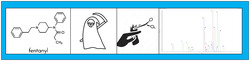

## 1 Introduction

Fentanyl and its derivatives (nonpharmaceutical fentanyl, NPFs) represent the largest group among synthetic opioids [1]. Fentanyls are highly lethal even at low doses, with nearly sudden deaths occurring mainly because of respiratory depression. Fentanyls‐related deaths have been reported in recreational use/abuse and therapeutic practice. Furthermore, fatalities from tampering with pharmaceutical products so that they can be smoked, snorted, injected, or taken orally have been reported [2]. In the United States, from 2013 to 2019, the age‐adjusted rate of deaths involving synthetic opioids increased by 1040% [3].

Since the mid‐2010s, adulterants such as xylazine, a veterinary drug worsening the hypotension and depression of the central nervous system and respiration caused by opiates, have been detected in an increasing number of unintentional overdose deaths involving opiates and/or fentanyl [4, 5]. Because of the intrinsic toxicity of xylazine, the search for it in biological fluids is of utmost importance.

From July 2019 and during the COVID‐19 pandemic (early 2020), substances included in the nitazenes opioid class, including isotonitazene and metonitazene, were identified for the first time in Europe and North America in the street drug supply and toxicological samples [6, 7]. Subsequently, numerous cases of opioid overdose deaths involving nitazenes have been reported, suggesting the spread of this class of substances among recreational drug users [8].

Taking into consideration the above evidence, the need to monitor the distribution of fentanyl, NPFs, nitazenes, and xylazine among the population represents a fundamental pursuit in forensic toxicology to understand their role in the overdose‐risk environment and, possibly to prevent synthetic opioid overdose fatalities.

In forensic toxicology, the use of hair analysis has gained attention over the years, especially for the retrospective investigation of chronic drug abuse and the unique ability of hair to serve as a long‐term storage site for xenobiotics, providing an extended diagnostic detection window (from weeks to months, depending on the length of the hair shaft) [9].

Although several papers have been published regarding the determination of synthetic opioids in hair [10, 11], only a few included nitazenes [12, 13], or post‐mortem samples [14]. Moreover, analytical methods for the determination of xylazine in hair are still lacking.

On this basis, the present study aimed to develop a novel UPLC‐MS/MS method for the detection and quantification of fentanyl, NPFs, nitazenes, and xylazine in post‐mortem hair samples. The method was successfully validated and applied to the determination of the target analytes in hair collected at autopsy (*n* = 250) to evaluate the prevalence of synthetic opioids and xylazine.

## 2 Materials and Methods

### 2.1 Standard Solutions and Reagents

Fentanyl, norfentanyl, β‐hydroxyfentanyl, acetylfentanyl, acetyl norfentanyl, despropionyl para‐fluorofentanyl, 4‐aminophenyl‐1‐phenethylpiperidine (4‐ANPP), carfentanil, norcarfentanil, ocfentanil, furanylfentanyl, and U‐47700 were obtained from Comedical (Trento, Italy) as certified standard methanolic solutions at concentrations between 0.02 and 0.05 mg/mL, whereas fentanyl‐D5, used as internal standard (IS), was purchased from Cerilliant (Round Rock, TX, USA). Xylazine and xylazine‐D6 were purchased as powder (10–100 mg) from Merck (Darmstadt, Germany), and a stock solution was obtained by diluting the powder in methanol to a final concentration of 1 mg/mL. Flunitazene, etodesnitazene, metonitazene, protonitazene, N‐piperidinyl etodesnitazene, and N‐pyrrolidino etodesnitazene were obtained from Comedical (Trento, Italy) as certified standard methanolic solutions at concentrations of 0.01 mg/mL. LC‐MS grade, methanol, acetonitrile, hydrochloric acid (HCl), and formic acid were purchased from VWR International (Radnor, PA, USA). Ultrapure Milli Q water was provided using the in‐house Purelab Chorus by Elga Veolia water purification system (High Wycombe, UK).

### 2.2 Hair Sample Preparation

Hair samples (50 mg) were placed in 100 mL glass Baker containing 40 mL of aqueous 3% Tween solution (*w/v*) and washed twice for 15 min by gently shaking. Hair was then dried overnight at room temperature and cut into small segments with scissors. After the addition of 10 μL of a mixture of fentanyl‐D5 and xylazine‐D6 (to a final concentration of 200 pg/mg and 5 ng/mg, respectively), hair was incubated with 1 mL of 0.1‐M HCl overnight at 45°C. Samples were neutralized with 230 μL of 0.1‐M NaOH and centrifuged at 4000 g for 5 min. The supernatant was eluted through a HyperSep Verify‐CX solid phase extraction (SPE, 130 mg) cartridge (Thermo Scientific, Milan, Italy). The cartridges were activated with 2‐mL methanol and equilibrated with 2‐mL phosphate buffer (pH 9) before loading the sample, sequentially washing with 2‐mL bidistilled water, 3‐mL 0.1‐M HCl, and 3.5‐mL methanol. The analytes were eluted with 2‐mL dichloromethane‐isopropanol mixture (80:20, *v/v*) with 2% ammonia solution. The eluate was dried under a nitrogen stream at 60°C and reconstituted in 50 μL of solvent A, before being injected into the LC‐MS/MS system.

### 2.3 Instrumentation and Analytical Conditions

Separations were performed on a model I‐Class ACQUITY UPLC system (Waters, Milford, MA, USA) using a Force Biphenyl column (2.1 × 50 mm, 1.8 μm, Restek Corporation, Bellefonte, PA, USA) and an UltraShield UHPLC PreColumn filter (0.2‐μm frit) kept at 45°C. Mobile phase A was composed of water and formic acid 0.1%, whereas mobile phase B consisted of acetonitrile. The injected samples were eluted with a linear gradient from 5% to 70% of solvent B, lasting 5 min. The column was washed with 90% of phase B and then the starting conditions were restored in 2 min and kept for 3 min to allow system re‐equilibration. The flow rate was set at 0.4 mL/min. The injection volume was 2 μL. The liquid chromatograph was coupled with an API 6500 QTrap mass spectrometer equipped with an IonDrive Turbo Spray V ion source (AB Sciex, Framingham, MA, USA). The instrument was operated in the positive‐ion mode with the following optimized parameters: curtain gas (nitrogen): 30 L/h; IonSpray Voltage: 5500 V; source temperature: 600°C; Ion Source Gas 1 and Gas 2 (air): 60 and 70 L/h, respectively. The analyses were performed in multiple reaction monitoring (MRM) mode using the optimized transitions and mass parameters reported in Table 1.

**TABLE 1:** Multiple‐reaction monitoring (MRMs) transitions, optimized mass parameters, and retention times of the studied compounds and their internal standards (quantifier transitions are in bold).

Analyte	Precursor ion (Da)	Daughter ion (Da)	DP (V)	EP (V)	ce (V)	CXP (V)	Retention time (min)
Fentanyl	337.2	**188.1**	44	6	31	12	2.85
		105.0	50	6	46	8	
Norfentanyl	233.0	**84.1**	48	7	25	9	1.75
		151.1	30	7	16	9	
β‐Hydroxy fentanyl	353.0	**335.1**	10	4	25	11	2.57
		186.1	10	4	36	17	
Acetyl fentanyl	323.0	**188.1**	16	4	31	11	2.54
		105.0	50	4	41	8	
Acetyl norfentanyl	219.0	**84.1**	41	8	24	9	1.38
		105.0	21	8	21	10	
Despropionyl para‐fluorofentanyl	299.1	**188.1**	16	5	25	11	2.91
		105.0	10	5	40	14	
4‐ANPP	280.9	**188.1**	19	6	25	19	2.84
		105.0	19	6	41	15	
Carfentanil	395.1	**335.1**	10	6	25	9	3.08
		363.4	27	6	19	9	
Norcarfentanil	290.9	**231.2**	26	4	18	16	1.92
		259.1	14	4	20	12	
Ocfentanil	371.2	**188.1**	20	5	31	13	2.53
		105.0	28	5	55	15	
Furanylfentanyl	375.2	**188.3**	38	4	50	20	3.05
		105.0	33	4	20	9	
U‐44700	330.2	**286.0**	46	5	24	24	2.72
		206.0	31	5	30	15	
Xylazine	221.0	**90.0**	33	10	30	13	2.06
		148.2	22	10	51	11	
Flunitazene	371.1	**252.2**	40	5	45	15	2.70
		108.8	23	5	75	17	
Etodesnitazene	352.2	**100.2**	35	4	25	15	2.03
		72.1	50	4	59	10	
Metonitazene	383.2	**100.0**	29	4	29	14	2.70
		310.0	18	4	33	18	
Protonitazene	411.2	**100.0**	23	4	29	14	3.35
		72.1	30	4	45	10	
N‐piperidynil etodesnitazene	409.3	**112.0**	40	4	32	12	3.07
		106.9	59	4	35	7	
N‐pyrrolidino etodesnitazene	395.2	**98.1**	33	4	29	12	2.92
		135.1	30	4	29	17	
Fentanyl‐D5 (IS)	342.5	**188.1**	44	6	31	12	2.84
		105.0	50	6	46	8	
Xylazine‐D6 (IS)	226.0	**90.0**	34	5	31	11	2.04
		153.2	56	5	42	10	

### 2.4 Method Validation

The method was validated according to the ANSI/ASB Standard 036, Standard Practices for Method Validation in Forensic Toxicology [15], in terms of interferences (selectivity), linearity, sensitivity (limit of detection [LOD] and limit of quantification [LOQ]), intraday and interday precision, accuracy (bias), matrix effect (ME) and extraction recovery (RE), carryover, and processed sample stability.

Selectivity was assessed by testing 20 different drug‐free (negative) hair samples spiked with and without IS to evaluate interferences from the matrix at the retention times of any of the transitions selected for the analysis of the compounds of interest.

To evaluate linearity, blank hair samples were fortified with different concentrations of the target analytes and then processed according to the procedure described in Section 2.2. Linearity was assessed on five different days within the expected concentration range. At least six different nonzero levels and five replicates per concentration in separate runs were tested. The calibration curves for the analytes were created by weighted regression analysis of the normalized peak areas (analyte area/IS area).

The limit of quantification (LOQ) for all the studied compounds was calculated as the lowest concentration of analyte that can be quantitatively determined with an acceptable (< 20%) precision and accuracy (bias).

The limit of detection (LOD) was assessed over multiple runs and expressed as the analyte concentrations associated with chromatographic peaks with a S/*N* ≥ 3.

Intraday and interday precision were calculated on five different days using five replicates of three chosen QC levels (low, medium, high) for each compound. The intraday assessments were expressed in terms of percent relative standard deviation (intraday or interday %RSD) and considered acceptable below 20%. The method's accuracy was expressed as intraday and interday bias, or percentage deviation from the expected value, within ±20%.

To assess the matrix effect (ME) the post‐extraction addition approach was used [16]. Briefly, different sets of samples were prepared, and the analyte peak areas of neat standards (set A) were compared with matrix samples fortified with neat standards after extraction (set B) or processing (set C). The matrix effect was analyzed on 10 different hair matrix sources in three replicates and evaluated at three concentration levels. In addition, the extraction recovery (RE) was also determined. ME and RE were calculated according to the following equations:ME (%) = (B/A) × 100; RE (%) = (C/B) × 100.

For the evaluation of analyte carryover, different blank matrix samples (*n* = 10) were analyzed immediately after the highest calibration point for each compound.

The processed sample stability was evaluated by reinjecting every 2 h low and high QCs kept at 10°C in the autosampler over an entire day of analysis. The average responses at each time interval were compared with the time zero responses, and the sample stability acceptance criteria was < 25%.

### 2.5 Analysis of Authentic Samples

Hair samples (*n* = 250) were obtained from all autopsy cases collected at the jurisdiction of the Jefferson County Coroner/Medical Examiner Office from January 2023 to March 2023.

The cases, which were anonimized by using an alphanumeric code, included drug overdose deaths, homicides, suicides, traffic accidents, and natural deaths.

The samples were stored at room temperature in their original collection kit until analyzed.

Hair analysis was carried out at the Unit of Forensic Medicine of the Dept. of Diagnostics and Public Health of the University of Verona. For all samples, proximal 3–5 cm were analyzed.

## 3 Results and Discussion

### 3.1 Method Validation

The method was shown to be selective since no interferences from the matrix or from the internal standards at the retention times of any of the studied compounds were observed.

Linearity was verified in the range of 3–10,000 pg/mg for fentanyl, norfentanyl, β‐hydroxyfentanyl, and between 9 and 10,000 pg/mg for 4‐ANPP; in the range of 3–1200 pg/mg for acetylfentanyl, acetyl norfentanyl, carfentanil, norcarfentanil, ocfentanil, furanylfentanyl, and U‐47700; between 18.8 and 1200 pg/mg for despropionyl para‐fluorofentanyl. Linearity for xylazine was evaluated in the range of 62.5–10,000 pg/mg. For nitazenes (namely flunitazene, etodesnitazene, metonitazene, protonitazene, N‐piperidinyl etodesnitazene, N‐pyrrolidino etodesnitazene), linearity was assessed in the range of 50–1600 pg/mg.

The LOQs for all the compounds corresponded with the above reported lowest nonzero calibrators.

The LODs were calculated to be 0.9 pg/mg for fentanyl, norfentanyl, β‐hydroxyfentanyl, acetylfentanyl, acetyl norfentanyl, carfentanil, norcarfentanil, ocfentanil, furanylfentanyl, and U‐47700; 2.7 pg/mg for 4‐ANPP, and 5.6 pg/mg for despropionyl para‐fluorofentanyl; 18.8 pg/mg for xylazine; and 15 pg/mg for flunitazene, etodesnitazene, metonitazene, protonitazene, N‐piperidinyl etodesnitazene, and N‐pyrrolidino etodesnitazene.

It is worth mentioning that the sensitivity of the method for the NPFs was comparable with other LC‐MS/MS published methods [11, 14, 17, 18].

As reported in Table 2, intraday and interday precision and bias for all the tested levels over the 5 days (five replicates of low, medium, high QC levels) for all the compounds were within the acceptable range of ±20%.

**TABLE 2:** Intraday and interday precision and accuracy (bias) experiments evaluated on five different days using five replicates of three QC levels (low, medium, high) for each compound.

Analyte	Levels (pg/mg)	Intraday‐1		Intraday‐2		Intraday‐3		Intraday‐4		Intraday‐5		Interday	
		RSD %	BIAS %	RSD %	BIAS %	RSD %	BIAS %	RSD %	BIAS %	RSD %	BIAS %	RSD %	BIAS %
Fentanyl	Low 9	11.4	10.9	18.7	12.7	10.4	8.7	0.3	10.6	6.2	7.7	9.4	10.1
	Middle 300	2.2	6.5	2.8	1.0	7.3	5.9	0.2	12	1.2	8.7	4.7	6.9
	High 1200	0.5	1.0	0.4	0.7	0.6	0.9	0.2	1.9	1.4	−1.7	1.4	0.6
Norfentanyl	Low 9	2.8	9.7	5.0	19.9	0.7	9.4	12.0	11.1	19.3	19.8	7.2	8.0
	Middle 300	2.4	9.0	3.7	15	2.5	14	3.6	13	1.2	8.7	3.7	12
	High 1200	0.2	−0.6	2.3	−2.7	0.6	−2.6	0.2	−0.9	1.4	−1.7	1.3	−1.7
β‐Hydroxy fentanyl	Low 9	8.5	3.1	9.9	19.0	5.7	2.1	1.6	18.8	3.4	−4.9	5.9	7.6
	Middle 300	1.5	1.6	7.6	6.6	1.5	11	11	10	14	12	9.0	8.3
	High 1200	0.2	−0.2	0.2	−1.1	1.0	−2.0	0.2	−1.4	0.6	−1.7	0.8	−1.3
Acetyl fentanyl	Low 9	9.0	18.6	8.5	19.3	19.2	5.2	2.6	2.3	19.7	−8.3	14.0	8.5
	Middle 300	0.3	11	6.1	15	14	15	12	13	15	12	11	13
	High 1200	0.2	−0.8	0.1	−1.3	0.1	−1.8	0.2	−1.6	0.1	−2.4	0.6	−1.6
Acetyl norfentanyl	Low 9	1.3	15.3	19.2	7.9	1.5	−1.9	3.2	2.3	3.5	−16.5	5.9	1.4
	Middle 300	0.7	8.5	2.4	14	0.5	6.7	2.5	9.2	0.1	15	3.7	11
	High 1200	0.3	−1.3	0.3	−3.0	0.1	−2.0	0.4	−0.8	1.1	−5.5	1.9	−2.5
Despropionyl para‐fluorofentanyl	Low 37.5	1.6	10	5.8	13	4.7	11	3.5	12	7.7	15	5.1	12
	Middle 300	12	−10	11	−12	11	−7.9	14	−10	13	−8.6	14	−10
	High 1200	0.2	1.3	0.2	1.0	0.3	1.1	0.2	0.8	0.4	1.4	0.3	1.1
4‐ANPP	Low 18.8	1.6	−0.9	10.6	10.9	3.3	−8.3	2.9	1.6	10.4	−3.5	5.9	0.1
	Middle 300	4.3	−7.2	0.9	6.9	10	0.3	5.8	2.5	1.8	−5.8	5.8	−0.6
	High 1200	0.1	0.3	0.1	−0.8	0.5	−0.5	0.4	−0.4	0.6	0.7	0.4	−0.1
Carfentanyl	Low 9	13.1	13.5	17.4	6.3	3.3	1.5	7.8	7	19.2	18.8	12.2	1.5
	Middle 300	0.5	0.2	2.5	0.2	0.2	2.7	0.1	5.3	0.7	6.8	1.2	3.0
	High 1200	0.1	0.4	0.9	−0.2	0.1	0.5	0.2	1.2	0.2	−1.1	0.4	0.2
Norcarfentanyl	Low 9	7.0	−9.1	12.8	2.2	5.0	8.7	10.4	19.3	3.2	19.6	7.8	−8.3
	Middle 300	4.9	11	10	11	3.9	7.3	1.1	15	12	11	7.4	11
	High 1200	0.3	−0.7	0.4	−2.4	12	−8.7	1.0	−3.4	0.2	−1.4	5.2	−3.3
Ocfentanyl	Low 9	19.6	−10.8	5.4	18.2	3.2	−2.2	8.3	19.7	5.8	19.8	8.2	−6.1
	Middle 300	6.1	13	8.8	7.8	0.9	8.2	3.2	15	5.1	9.3	5.2	11
	High 1200	0.3	−1.9	10	−11	11	−13	0.6	−2.4	5.1	−6.8	7.3	−7.2
Furanyl fentanyl	Low 9	15.5	−18.7	19.3	−4.6	9.2	8.4	2.8	−5.8	8.5	−1.8	12.8	−4.5
	Middle 300	6.0	13	14	13	2.9	15	5.5	13	0.3	10	7.5	7.5
	High 1200	0.3	−1.0	0.4	−1.2	0.4	−0.6	0.1	−0.7	0.7	−1.9	0.4	0.6
U‐44700	Low 9	5.1	12.5	7.5	19.3	1.2	18.2	2.4	18.5	4.7	2.6	3.8	−4.3
	Middle 300	4.3	−5.6	3.3	9.9	0.2	6.8	9.7	6.5	5.5	5.6	5.6	6.9
	High 1200	1.2	2.1	1.7	−2.4	0.1	0.6	0.4	2.5	12	−15	8.4	−2.6
Xylazine	Low 125	1.9	−0‐2	1.9	−0.8	3	−0.6	2.1	−0.9	1	5.3	2	0.5
	Middle 500	0.5	6.5	0.3	7.0	0.2	4.4	0.1	2.7	1.8	−1.8	3.5	3.8
	High 2000	0.2	0.7	0.1	1.0	0.2	−1.0	0.1	1.3	0.6	0.3	0.3	0.9
Flunitazene	Low 100	1.0	15	2.2	14	0.9	15	12	14	3.7	13	5.7	14
	Middle 400	1.6	−6.8	0.9	−13	3.5	−10	1.2	−3.5	2.4	−7.6	4.5	−8.4
	High 1600	0.2	0.5	0.1	0.8	0.1	0.6	0.1	1.4	0.2	0.6	0.2	0.6
Etodesnitazene	Low 100	10.3	4.0	4.6	−0.5	8.3	4.0	12	14	6.7	9.6	9.0	6.3
	Middle 400	3.4	11	9.8	10	3.8	9.5	0.1	2.7	1.8	−1.8	11	8.2
	High 1600	0.2	−0.6	2.3	1.4	0.3	−0.6	0.5	2.2	1.5	1.7	1.6	0.8
Metonitazene	Low 100	8.1	10	6.5	14	3.3	15	6.1	6.5	3.5	−7.2	9.5	7.9
	Middle 400	3.1	5.6	1.1	6.1	1.8	1.4	5.8	15	3.2	6.3	5.5	7.0
	High 1600	1.8	1.1	0.8	0.6	0.1	−0.2	0.4	−0.7	0.1	−0.3	1.0	0.1
Protonitazene	Low 100	9.6	12	1.7	15	10	14	14	13	11	14	10	14
	Middle 400	9.0	12	0.4	−0.1	3.8	7.2	10	4.6	1.0	6.7	6.4	5.8
	High 1600	0.2	2.3	0.2	3.2	1.6	0.8	0.5	3.4	0.2	3.3	1.2	2.6
N‐piperidynil etodesnitazene	Low 100	8.4	14	2.0	11	0.5	15	12	15	7.7	15	13	8.2
	Middle 400	0.2	−14	0.2	−7.6	14	−4.2	4.5	−12	0.1	−9.9	6.8	−9.5
	High 1600	0.2	0.6	0.1	0.3	0.7	0.2	0.2	0.5	1.6	1.7	0.8	0.7
N‐pyrrolidino etodesnitazene	Low 100	3.6	15	10	11	8.0	11	6.2	12	11	10	8.2	12
	Middle 400	5.9	−15	8.8	−14	0.4	−14	1.0	−13	0.2	−15	4.8	−14
	High 1600	0.3	0.7	0.1	1.0	0.2	0.8	0.1	0.7	0.2	0.8	0.2	0.8

The mean matrix effect of the analytes tested at two different concentration levels were within the acceptable range of 75%–125% for all the analytes, except for nitazenes, which showed matrix effects values outside the range of acceptability mainly for the high QC level (Table 3). For this reason, for this specific class of analytes, the method can be regarded as semiquantitative.

**TABLE 3:** Matrix effect (ME%) and extraction recovery (RE%) evaluated at the selected QCs.

Analyte	Levels (pg/mg)	Matrix effect (ME)	Extraction recovery (RE)
		75%–125%	> 50%
Fentanyl	QC low 37.5	89.3	94.2
	QC high 1200	94.2	94.0
Norfentanyl	QC low 37.5	83.9	108
	QC high 1200	76.3	52.4
β‐Hydroxy fentanyl	QC low 37.5	77.0	115
	QC high 1200	86.4	80.7
Acetyl fentanyl	QC low 37.5	113	81
	QC high 1200	99	99
Acetyl norfentanyl	QC low 37.5	94.2	115
	QC high 1200	74.3	52.6
Despropionyl para‐fluorofentanyl	QC low 37.5	88.1	107
	QC high 1200	85.6	95.5
4‐ANPP	QC low 37.5	124	107
	QC high 1200	121	84.3
Carfentanyl	QC low 37.5	82.9	118
	QC high 1200	92.9	111
Norcarfentanyl	QC low 37.5	86.0	100
	QC high 1200	75.7	57.6
Ocfentanyl	QC low 37.5	75.8	114
	QC high 1200	76.8	89.9
Furanyl fentanyl	QC low 37.5	88.7	108
	QC high 1200	106	113
U‐44700	QC low 37.5	88.7	106
	QC high 1200	83.5	115
Xylazine	QC low 125	85.0	105
	QC high 2000	94.2	93.9
Flunitazene	QC low 100	116	105
	QC high 1600	>125	122
Etodesnitazene	QC low 100	115	117
	QC high 1600	124	101
Metonitazene	QC low 100	83.2	75.8
	QC high 1600	>125	96.6
Protonitazene	QC low 100	98.6	>125
	QC high 1600	>125	107
N‐piperidynil etodesnitazene	QC low 100	119	112
	QC high 1600	>125	102
N‐pyrrolidino etodesnitazene	QC low 100	88.9	100
	QC high 1600	>125	125

The extraction recoveries were consistently higher than 50% (Table 3).

No evidence of carryover was seen by injecting blank samples after high‐concentration samples. Moreover, good sample stability was verified for the low and high QCs (within ±25% of the target concentration).

**FIGURE 1:**
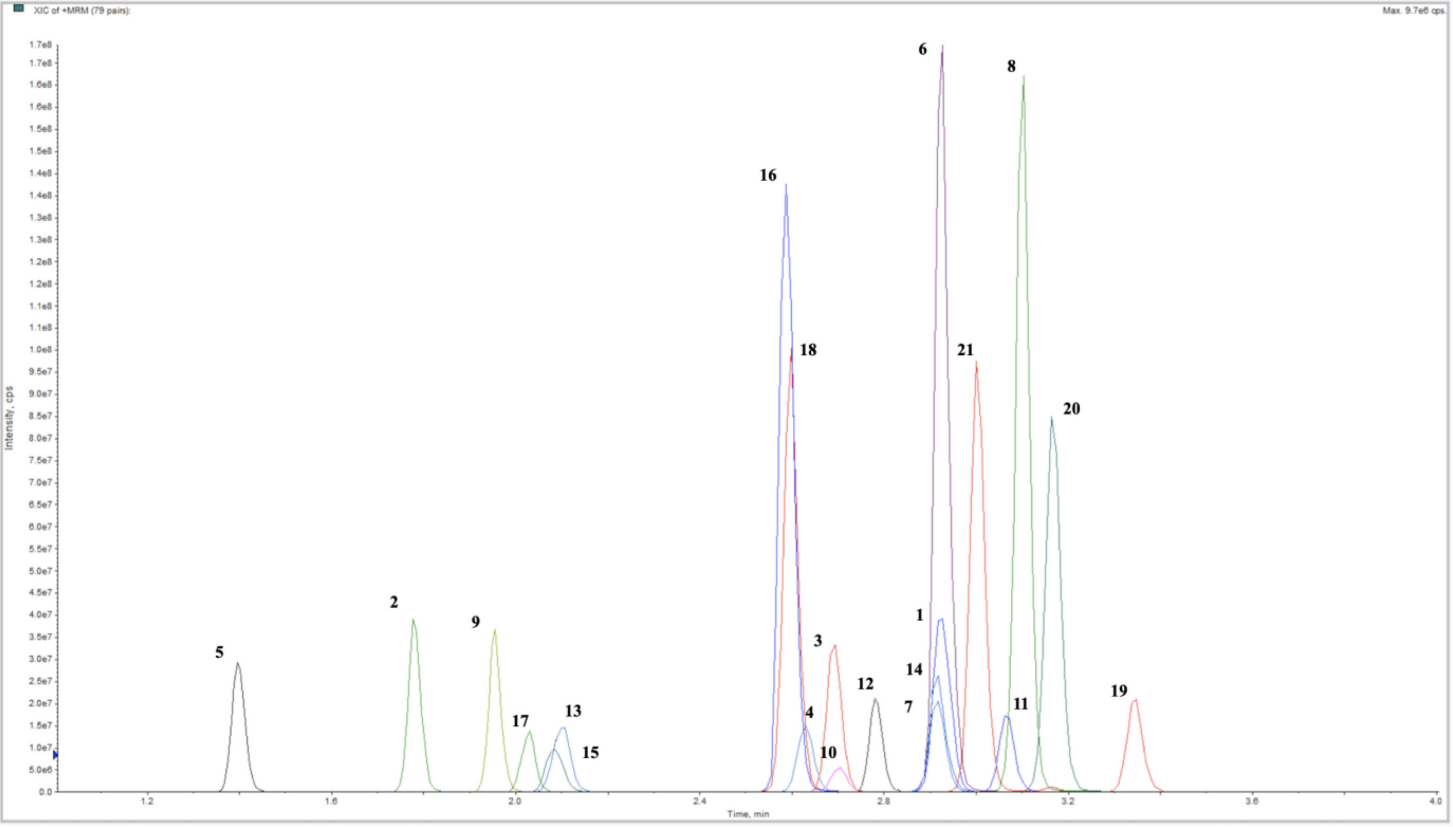
Extract ion chromatograms of a mixture of the studied analytes at a concentration of 1000 pg/mg (**1**: fentanyl; **2**: norfentanyl; **3**: β‐hydroxyfentanyl; **4**: acetylfentanyl; **5**: acetyl norfentanyl; **6**: despropionyl para‐fluorofentanyl; **7**: 4‐ANPP; **8**: carfentanil; **9**: norcarfentanil; **10**: ocfentanil; **11**: furanylfentanyl; **12**: U‐44700; **13**: xylazine; **14**: fentanyl‐D5; **15**: xylazine‐D6; **16**: flunitazene; **17**: etodesnitazene; **18**: metodesnitazene; **19**: protonitazene; **20**: N‐piperidynil etodesnitazene; **21**: N‐pyrrolidino etodesnitazene).

Under the validated analytical conditions described above, the separation of all the studied compounds was obtained in less than 5 min. A representative chromatogram of a mixture of the compounds, each at an individual concentration of 1000 pg/mg, is depicted in Figure 1.

### 3.2 Analysis of Authentic Samples

Among the studied population, *n* = 129 of the analyzed hair samples (52%) tested positive for fentanyl either alone or with its main metabolites (norfentanyl, β‐hydroxyfentanyl, and 4‐ANPP), and/or NPFs. It is worth mentioning that one of the 250 cases tested positive for protonitazene, a synthetic opioid recently spread across the illicit market [7, 8].

The prevalence of synthetic opioid‐positive cases was significantly higher than anticipated considering that the cause of death in many of these cases was not related to synthetic opioids (53 out of 130 cases; 40.8%). Actually, this evidence is not surprising since the hair analysis provides a retrospective picture of the toxicological habits of the subjects, which could not be related to the cause of death. The data concerning the cause of death of the 130 synthetic opioid‐positive cases are provided as Supporting Information.

Fentanyl hair concentrations ranged from 4 pg/mg to a concentration higher than the ULOQ (mean value 1697 pg/mg, median 194 pg/mg). It is noteworthy that nearly 30% of positive cases (37/130) showed a fentanyl hair concentration higher that 1000 pg/mg, whereas only 10% showed fentanyl hair concentration lower than 10 pg/mg (13/130). These data confirmed that the calibration ranges chosen for the method validation were appropriate to the studied population living in a fentanyl high spread environment.

Regarding fentanyl metabolites, norfentanyl was detected in *n* = 69 cases of the samples positive for fentanyl and then in a lower percentage than that found in another similar research study performed on hair samples from living people [12] (53% vs. 85%).

On the other hand, consistently with the just mentioned study, in all the norfentanyl positive cases, but three, fentanyl was detected.

Norfentanyl concentrations ranged from the LOQ to concentrations higher than the ULOQ (mean value 428 pg/mg, median 24 pg/mg). β‐Hydroxyfentanyl was present in *n* = 35 hair, with concentrations from 3.34 to 2350 pg/mg (mean value 380 pg/mg, median 116 pg/mg). 4‐ANPP was detected in *n* = 70 cases, corresponding to about 54% of cases in which fentanyl was measured. 4‐ANPP concentrations were in the range 9–8401 pg/mg (mean value 516 pg/mg, median 96 pg/mg). Both β‐hydroxyfentanyl and 4‐ANPP were detected only in hair also containing fentanyl. It should be noted that 4‐ANPP is both a metabolite of fentanyl and a product of illicit manufacture.

**FIGURE 2:**
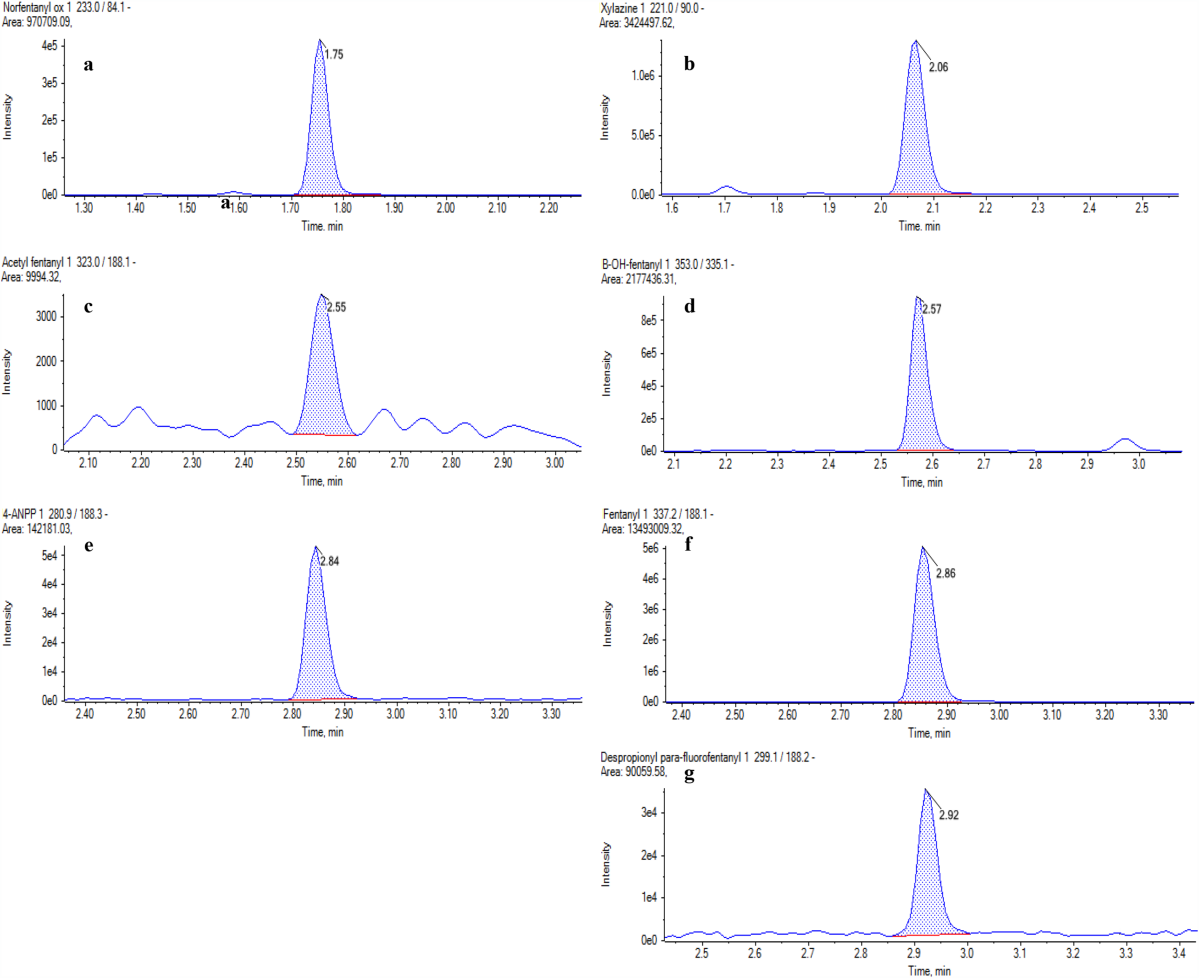
Extract ion chromatogram of a real hair sample with a detectable amount of the following analytes: (a) norfentanyl (235 pg/mg); (b) xylazine (2540 pg/mg); (c) acetylfentanyl (5 pg/mg); (d) β‐hydroxyfentanyl (296 pg/mg); (e) 4‐ANPP (187 pg/mg); (f) fentanyl (4170 pg/mg); (g) despropionyl para‐fluorofentanyl (22 pg/mg).

In 23 cases, in addition to fentanyl, acetylfentanyl and/or despropionyl para‐fluorofentanyl were detected. In particular, 11 cases contained fentanyl, acetylfentanyl and despropionyl para‐fluorofentanyl. Seven cases tested positive for both fentanyl and despropionyl para‐fluorofentanyl, whereas five cases contained fentanyl and acetylfentanyl. The concentrations of acetylfentanyl and despropionyl para‐fluorofentanyl ranged from 3 to 431 pg/mg (mean value 104 pg/mg, median 22 pg/mg) and from 5 to 1385 pg/mg (mean value 129 pg/mg, median 25 pg/mg), respectively. An extract ion chromatogram of a real case sample with detectable amounts of the previous mentioned analytes was reported in Figure 2.

To the best of our knowledge, the present study concerning synthetic opioid hair testing in post‐mortem cases is the first one including a significant number of subjects (*n* = 250). Indeed, the other studies reported in literature tested overall only 38 cases with 15 positivity [14, 19, 20, 21, 22, 23, 24, 25, 26]. On the other hand, fentanyl and NPFs mean concentrations retrieved in the present population were up to two orders of magnitude higher than those reported in the cited papers. This great difference may be related to the different geographical area (United States vs. Europe) of the studied populations. Indeed, only one of the cited papers, which concerns the US population, reported fentanyl concentrations similar to those of the present study [19].

Forty‐eight cases out of 130 positive for synthetic opioids showed the presence of xylazine in a wide range of concentrations (from LOQ to the ULOQ, with a median concentration of 307 pg/mg and a mean concentration of 1358 pg/mg), confirming the spreading use of this compound as adulterant of NPFs.

**FIGURE 3:**
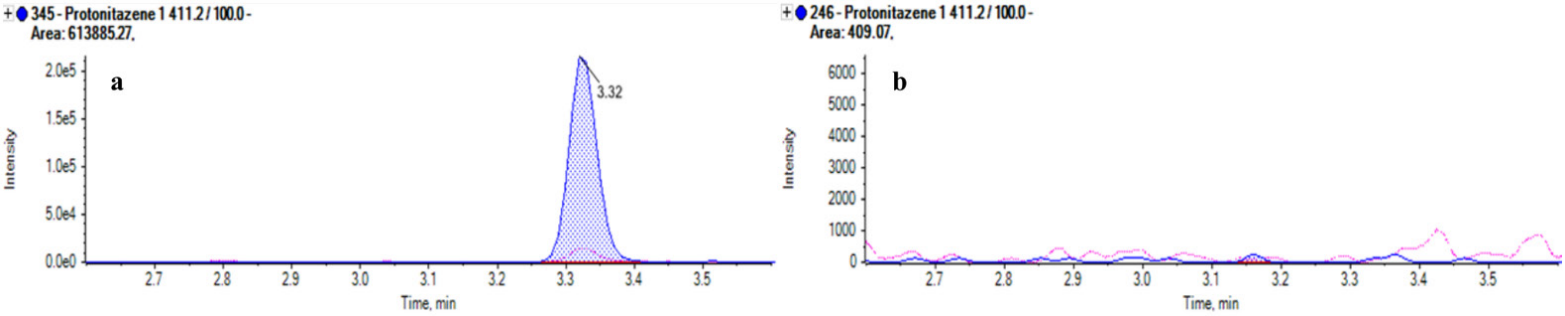
Extract ion chromatogram of a real hair sample with a detectable amount of protonitazene (a: 24 pg/mg) and a blank sample (b).

Nitazenes were absent, with the exception of the already mentioned case in which protonitazene was detected (Figure 3) in an extremely low concentration (between LOD and LOQ). This case was collected from a subject with a history of heavy opioid abuse found dead in his home, who eventually died from a protonitazene overdose. The nitazenes are more potent than many of the NPFs, and therefore, the higher LOD of the nitazenes in this method may have resulted in some nitazenes not being detected because of the low levels of these drugs.

To our knowledge, this is the first time that xylazine and protonitazene were detected in hair.

## 4 Conclusions

The UPLC‐MS/MS method developed for the detection and quantification of fentanyls, xylazine, and for semiquantitative evaluation of nitazenes in human hair samples was fully validated and suitable for rapid chromatographic separation and sensitive detection of the studied compounds in a complex matrix, such as post‐mortem hair.

The application of the method to real postmortem cases showed significant exposure to fentanyl, analogs, and adulterants in the studied population, confirming the usefulness of hair testing to retrospectively investigate chronic exposure to synthetic opioids.

In the studied population, fentanyl, consistent with the epidemiological data on the spread of NPFs, was the most frequently detected synthetic opioid, even though acetylfentanyl and despropionyl para‐fluorofentanyl were also present in many samples.

The developed method allowed for the detection of xylazine in hair providing critical information about the xylazine emerging threat.

## Conflicts of Interest

The authors declare no conflicts of interest.

## Supporting information


**Data S1:** Supplementary information

## Data Availability

The data analyzed in the present study are available from the corresponding author upon reasonable request.
