# Supplementary material for: Retrospective Evaluation of Novel Synthetic Opioids and Xylazine Chronic Intake by Post‐Mortem Hair Testing
Source: Drug Test Anal. 2025 Jan 21;17(9):1516–27. doi: 10.1002/dta.3852 (PMC12401624; doi:10.1002/dta.3852)
Supplement: Supplementary file 1 — Data S1: Supplementary information [file DTA-17-1516-s001.docx]

| **Case Number** | **Hair analysis** | **Cause of death** |
| --- | --- | --- |
| **1** | Fentanyl = 43.1 pg/mg | Ethanol, fentanyl, cocaine toxicity |
| **2** | Fentanyl = 201 pg/mg  Norfentanyl = 980 pg/mg  ß-hydroxy fentanyl < LOQ  4-ANPP = 31.2 pg/mg | Fentanyl toxicity |
| **3** | Fentanyl > 10,000 pg/mg  Norfentanyl = 2420 pg/mg  Acetyl fentanyl = 9.13 pg/mg  ß-hydroxy fentanyl = 2,340 pg/mg  Despropionyl para-fluorofentanyl = 582 pg/mg  4-ANPP = 31.2 pg/mg  Xylazine = 9,800 pg/mg | Fentanyl Toxicity |
| **4** | Fentanyl = 6.66 pg/mg | Fentanyl and ethanol toxicity |
| **5** | Fentanyl = 1,070 pg/mg  Norfentanyl = 37.7 pg/mg  ß-hydroxy fentanyl = 26.5 pg/mg  4-ANPP = 143 pg/mg  Xylazine = 420 pg/mg | Heroin toxicity |
| **6** | Fentanyl = 40.4 pg/mg  Norfentanyl < LOQ  4-ANPP = 22.3 pg/mg | Cocaine and methamphetamine toxicity |
| **7** | Fentanyl = 780 pg/mg  Norfentanyl < LOQ  4-ANPP = 254 pg/mg | Cocaine toxicity |
| **8** | Fentanyl = 2,170 pg/mg  Norfentanyl < LOQ  Despropionyl para-fluorofentanyl = 320 pg/mg  4-ANPP = 291 pg/mg | Fentanyl toxicity |
| **9** | Fentanyl = 4,170 pg/mg  Norfentanyl = 235 pg/mg  Acetyl fentanyl = 4.58 pg/mg  ß-hydroxy fentanyl = 296 pg/mg  Despropionyl para-fluorofentanyl = 22 pg/mg  4-ANPP = 187 pg/mg  Xylazine = 2540 pg/mg | Fentanyl and methamphetamine toxicity |
| **10** | Fentanyl = 1,000 pg/mg  Norfentanyl < LOQ  4-ANPP = 192 pg/mg  Xylazine = 140 pg/mg | Fentanyl, cocaine, methamphetamine toxicity |
| **11** | Fentanyl = 57.6 pg/mg | Fentanyl and cocaine toxicity |
| **12** | Fentanyl = 47.8 pg/mg | Gunshot wound of head |
| **13** | Fentanyl = 105 pg/mg | Fentanyl toxicity |
| **14** | Fentanyl = 139 pg/mg | Fentanyl and cocaine toxicity |
| **15** | Fentanyl = 450 pg/mg  4-ANPP = 225 pg/mg | Fentanyl and dextromethorphan toxicity |
| **16** | Fentanyl = 27.8 pg/mg | Fentanyl and methamphetamine toxicity |
| **17** | Fentanyl = 2,270 pg/mg  Norfentanyl < LOQ  4-ANPP = 127 pg/mg  Xylazine = 240 pg/mg | Fentanyl and cocaine toxicity |
| **18** | Fentanyl = 53.3 pg/mg  Norfentanyl < LOQ | Fentanyl, cocaine, ethanol toxicity |
| **19** | Fentanyl = 195 pg/mg  Norfentanyl < LOQ  4-ANPP = 48.1 pg/mg  Xylazine = 140 pg/mg | Blunt force injuries |
| **20** | Fentanyl = 448 pg/mg  Norfentanyl = 3,810 pg/mg  ß-hydroxy fentanyl = 3.34 pg/mg  Despropionyl para-fluorofentanyl = 27.9 pg/mg  4-ANPP = 99.7 pg/mg  Xylazine = 530 pg/mg | Fentanyl and cocaine toxicity |
| **21** | Fentanyl > 10,000 pg/mg  Norfentanyl = 2,210 pg/mg  Acetyl fentanyl = 5.22 pg/mg  ß-hydroxy fentanyl = 1,520 pg/mg  Despropionyl para-fluorofentanyl = 35 pg/mg  4-ANPP = 2,720 pg/mg  Xylazine > 10,000 pg/mg | Fentanyl toxicity |
| **22** | Fentanyl = 63.9 pg/mg  Norfentanyl < LOQ | Fentanyl, cocaine, methamphetamine toxicity |
| **23** | Fentanyl = 472 pg/mg | Methamphetamine toxicity |
| **24** | Fentanyl = 178 pg/mg  Norfentanyl < LOQ  Xylazine = 80 pg/mg | Ruptured abdominal aortic aneurysm |
| **25** | Fentanyl = 3.72 pg/mg | Stab wound of torso |
| **26** | Fentanyl = 1,160 pg/mg  Norfentanyl < LOQ  ß-hydroxy fentanyl < LOQ  4-ANPP = 389 pg/mg  Xylazine = 190 pg/mg | Methamphetamine and fentanyl toxicity |
| **27** | Fentanyl = 194 pg/mg  4-ANPP = 50.2 pg/mg  Xylazine = 210 pg/mg | Fentanyl toxicity |
| **28** | Fentanyl = 345 pg/mg  Norfentanyl < LOQ  Despropionyl para-fluorofentanyl = 66.7 pg/mg  4-ANPP = 123 pg/mg  Xylazine = 210 pg/mg | Methamphetamine, cocaine, fentanyl toxicity |
| **29** | Fentanyl = 7.35 pg/mg | Undetermined |
| **30** | Fentanyl = 145 pg/mg | Congestive heart failure |
| **31** | Fentanyl > 10,000 pg/mg  Norfentanyl = 1,850 pg/mg  Acetyl fentanyl = 225 pg/mg  ß-hydroxy fentanyl = 900 pg/mg  Despropionyl para-fluorofentanyl = 112 pg/mg  4-ANPP = 220 pg/mg  Xylazine = 1,500 pg/mg | Gunshot wound of the left tight |
| **32** | Fentanyl = 40 pg/mg | Fentanyl, methamphetamine, cocaine toxicity |
| **33** | Fentanyl = 16.8 pg/mg | Asphyxia by hanging |
| **34** | Fentanyl = 4,380 pg/mg  4-ANPP = 164 pg/mg | Gunshot wound of head |
| **35** | Fentanyl = 110 pg/mg  Norfentanyl < LOQ  4-ANPP = 56.8 pg/mg  Xylazine = 90 pg/mg | Fentanyl and methamphetamine toxicity |
| **36** | Fentanyl = 224 pg/mg  Norfentanyl < LOQ  4-ANPP = 61.5 pg/mg  Xylazine = 270 pg/mg | Methamphetamine and fentanyl toxicity |
| **37** | Fentanyl = 5.69 pg/mg  Xylazine = 80 pg/mg | Gunshot wound of back |
| **38** | Fentanyl = 1,260 pg/mg  Norfentanyl = 23.6 pg/mg  4-ANPP = 110 pg/mg | Morphine toxicity |
| **39** | Fentanyl = 86.8 pg/mg  4-ANPP = 30.1 pg/mg | Blunt force injuries |
| **40** | Fentanyl = 480 pg/mg  Norfentanyl < LOQ  4-ANPP = 55.8 pg/mg  Xylazine = 90 pg/mg | Blunt force injuries |
| **41** | Fentanyl > 10,000 pg/mg  Norfentanyl = 2,740 pg/mg  Acetyl fentanyl = 4.27 pg/mg  ß-hydroxy fentanyl = 2,350 pg/mg  Despropionyl para-fluorofentanyl = 24.7 pg/mg  4-ANPP = 401 pg/mg  Xylazine = 380 pg/mg | Hemopericardium |
| **42** | Fentanyl = 9,940 pg/mg  Norfentanyl > 10,000 pg/mg  Acetyl fentanyl = 20.3 pg/mg  ß-hydroxy fentanyl = 1,250 pg/mg  4-ANPP = 249 pg/mg  Xylazine = 4,170 pg/mg | Fentanyl and heroin toxicity |
| **43** | Fentanyl = 56.3 pg/mg  Norfentanyl < LOQ  4-ANPP = 26.5 pg/mg  Xylazine = 110 pg/mg | Fentanyl toxicity |
| **44** | Fentanyl = 93.8 pg/mg  ß-hydroxy fentanyl < LOQ  4-ANPP = 60.5 pg/mg | Complications of heart failure |
| **45** | Fentanyl = 28.8 pg/mg  Norfentanyl < LOQ  4-ANPP = 15.7 pg/mg  Xylazine = 120 pg/mg | Chronic substance use |
| **46** | Fentanyl < LOQ | Gunshot wound of head |
| **47** | Fentanyl = 6,750 pg/mg  Norfentanyl = 307 pg/mg  ß-hydroxy fentanyl = 650 pg/mg  Despropionyl para-fluorofentanyl = 30.2 pg/mg  4-ANPP = 227 pg/mg  Xylazine = 890 pg/mg | Methamphetamine and fentanyl toxicity |
| **48** | Fentanyl = 3,310 pg/mg  Norfentanyl = 7.9 pg/mg  Acetyl fentanyl < LOQ  Despropionyl para-fluorofentanyl = 34.1 pg/mg  4-ANPP = 1,880 pg/mg  Xylazine = 270 pg/mg | Fentanyl toxicity |
| **49** | Fentanyl < LOQ | Acetaminophen toxicity |
| **50** | Fentanyl = 337 pg/mg  Norfentanyl < LOQ  ß-hydroxy fentanyl < LOQ  4-ANPP = 109 pg/mg  Xylazine = 90 pg/mg | Methamphetamine, cocaine, fentanyl toxicity |
| **51** | Fentanyl = 7,520 pg/mg  Norfentanyl = 1,290 pg/mg  Acetyl fentanyl = 23.9 pg/mg  ß-hydroxy fentanyl = 740 pg/mg  4-ANPP = 550 pg/mg  Xylazine = 1040 pg/mg | Fentanyl toxicity |
| **52** | Fentanyl = 9.34 pg/mg | Blunt force injuries |
| **53** | Fentanyl < LOQ | Hydrocodone and ethanol toxicity |
| **54** | Fentanyl = 8,400 pg/mg  Norfentanyl = 102 pg/mg  ß-hydroxy fentanyl < LOQ  4-ANPP = 122 pg/mg  Xylazine = 2,700 pg/mg | Undetermined |
| **55** | Fentanyl = 29 pg/mg | Hypertensive heart disease |
| **56** | Fentanyl = 144 pg/mg  Norfentanyl = 21 pg/mg | Fentanyl and cocaine toxicity |
| **57** | Fentanyl = 3,138 pg/mg  Norfentanyl = 180 pg/mg  ß-hydroxy fentanyl = 132 pg/mg  Despropionyl para-fluorofentanyl < LOQ  4-ANPP = 266 pg/mg  Xylazine = 487 pg/mg | Fentanyl toxicity |
| **58** | Fentanyl = 70 pg/mg  Norfentanyl = 43 pg/mg  ß-hydroxy fentanyl = 4 pg/mg  4-ANPP < LOQ | Fentanyl and ethanol toxicity |
| **59** | Fentanyl = 71 pg/mg  Norfentanyl = 17 pg/mg  4-ANPP < LOQ | Fentanyl toxicity |
| **60** | Fentanyl = 4 pg/mg | Fentanyl toxicity |
| **61** | Xylazine = 4135 pg/mg | Xylazine toxicity |
| **62** | Fentanyl = 212 pg/mg  ß-hydroxy fentanyl = 25 pg/mg  Despropionyl para-fluorofentanyl < LOQ  Xylazine = 78 pg/mg | Fentanyl and ethanol toxicity |
| **63** | Fentanyl = 7,677 pg/mg  Norfentanyl = 471 pg/mg  ß-hydroxy fentanyl = 436 pg/mg  Despropionyl para-fluorofentanyl < LOQ  4-ANPP = 374 pg/mg  Xylazine = 344 pg/mg | Fentanyl and methamphetamine toxicity |
| **64** | Fentanyl > 10,000 pg/mg  Norfentanyl = 2,054 pg/mg  ß-hydroxy fentanyl = 55 pg/mg  Despropionyl para-fluorofentanyl = 41 pg/mg  4-ANPP = 2,116 pg/mg  Xylazine = 6,385 pg/mg | Multiple gunshot wounds |
| **65** | Fentanyl = 8 pg/mg | Obesity cardiomyopathy |
| **66** | Fentanyl = 133 pg/mg  Norfentanyl = 48 pg/mg  ß-hydroxy fentanyl = 13 pg/mg  4-ANPP = 20 pg/mg  Xylazine = 120 pg/mg | Fentanyl toxicity |
| **67** | Fentanyl = 15 pg/mg | Arteriosclerotic cardiovascular disease |
| **68** | Fentanyl = 969 pg/mg  ß-hydroxy fentanyl = 28 pg/mg | Fentanyl toxicity |
| **69** | Fentanyl = 395 pg/mg  Norfentanyl = 51 pg/mg  4-ANPP = 180 pg/mg | Methamphetamine and fentanyl toxicity |
| **70** | Fentanyl = 1,258 pg/mg  Norfentanyl = 392 pg/mg  ß-hydroxy fentanyl = 96 pg/mg  4-ANPP = 27 pg/mg  Xylazine = 169 pg/mg | Blunt force injuries |
| **71** | Fentanyl = 223 pg/mg  Norfentanyl = 83 pg/mg  4-ANPP = 25 pg/mg  Xylazine = 191 pg/mg | Blunt force injuries |
| **72** | Fentanyl = 129 pg/mg  Norfentanyl = 19 pg/mg  4-ANPP = 48 pg/mg | Fentanyl and cocaine toxicity |
| **73** | Fentanyl = 320 pg/mg  Norfentanyl = 25.8 pg/mg | Fentanyl and cocaine toxicity |
| **74** | Fentanyl > 10,000 pg/mg  Norfentanyl = 374 pg/mg  Acetyl fentanyl = 424 pg/mg  ß-hydroxy fentanyl = 248 pg/mg  Despropionyl para-fluorofentanyl = 19 pg/mg  4-ANPP = 3,543 pg/mg  Xylazine = 426 pg/mg | Gunshot wound of head |
| **75** | Fentanyl > 10,000 pg/mg  Norfentanyl = 1,210 pg/mg  Acetyl fentanyl = 247 pg/mg  ß-hydroxy fentanyl = 448 pg/mg  Despropionyl para-fluorofentanyl = 24 pg/mg  4-ANPP = 9,926 pg/mg  Xylazine = 1,665 pg/mg | Gunshot wound of head |
| **76** | Fentanyl = 1,845 pg/mg  Norfentanyl = 464 pg/mg  ß-hydroxy fentanyl = 135 pg/mg  4-ANPP = 58 pg/mg  Xylazine = 808 pg/mg | Methamphetamine and fentanyl toxicity |
| **77** | Fentanyl = 9 pg/mg | Gunshot wound of head |
| **78** | Fentanyl = 267 pg/mg  Norfentanyl = 18 pg/mg  4-ANPP = 25 pg/mg | Methamphetamine, fentanyl, heroin toxicity |
| **79** | Fentanyl = 2,989 pg/mg  Norfentanyl = 867 pg/mg  ß-hydroxy fentanyl = 182 pg/mg  4-ANPP = 75 pg/mg | Fentanyl toxicity |
| **80** | Fentanyl > 10,000 pg/mg  Norfentanyl = 1,851 pg/mg  ß-hydroxy fentanyl = 837 pg/mg  Despropionyl para-fluorofentanyl = 28 pg/mg  4-ANPP = 4,497 pg/mg  Xylazine = 5,273 pg/mg | Fentanyl toxicity |
| **81** | Fentanyl = 57 pg/mg  Norfentanyl = 18 pg/mg  4-ANPP < LOQ  Xylazine = 111 pg/mg | Methamphetamine and fentanyl toxicity |
| **82** | Fentanyl = 118 pg/mg  Norfentanyl = 27.5 pg/mg  4-ANPP = 15 pg/mg | Methamphetamine and fentanyl toxicity |
| **83** | Fentanyl = 9,957 pg/mg  Norfentanyl = 637 pg/mg  Acetyl fentanyl = 111 pg/mg  ß-hydroxy fentanyl = 1,054 pg/mg  Despropionyl para-fluorofentanyl < LOQ  4-ANPP = 916 pg/mg  Xylazine = 1,048 pg/mg | Gunshot wound of right hip |
| **84** | Fentanyl = 291 pg/mg  4-ANPP = 33 pg/mg | Fentanyl toxicity |
| **85** | Fentanyl = 2,900 pg/mg  Norfentanyl = 742 pg/mg  Acetyl fentanyl = 10 pg/mg  ß-hydroxy fentanyl = 436 pg/mg  4-ANPP = 159 pg/mg  Xylazine = 543 pg/mg | Fentanyl toxicity |
| **86** | Fentanyl = 140 pg/mg  Norfentanyl = 46 pg/mg  Acetyl fentanyl = 12 pg/mg  ß-hydroxy fentanyl = 38 pg/mg  Despropionyl para-fluorofentanyl < LOQ  4-ANPP = 19 pg/mg | Fentanyl and methamphetamine toxicity |
| **87** | Fentanyl = 295 pg/mg  Norfentanyl = 21 pg/mg  ß-hydroxy fentanyl = 10 pg/mg  4-ANPP = 22 pg/mg  Xylazine < LOQ | Fentanyl and xylazine toxicity |
| **88** | Fentanyl = 445 pg/mg  Norfentanyl = 40 pg/mg  ß-hydroxy fentanyl = 30 pg/mg  4-ANPP = 35 pg/mg  Xylazine = 229 pg/mg | Fentanyl toxicity |
| **89** | Fentanyl = 28 pg/mg | Cocaine toxicity |
| **90** | Fentanyl = 330 pg/mg  Norfentanyl = 14 pg/mg  4-ANPP < LOQ | Fentanyl, hydrocodone, cocaine toxicity |
| **91** | Fentanyl = 56 pg/mg  Norfentanyl = 10 pg/mg  4-ANPP = 9 pg/mg | Gunshot wound of back |
| **92** | Fentanyl = 79 pg/mg  Norfentanyl = 28 pg/mg | Fentanyl, tramadol, ethanol, cocaine toxicity |
| **93** | Fentanyl = 1,830 pg/mg  Norfentanyl = 60 pg/mg  4-ANPP = 715 pg/mg  Xylazine < LOQ | Cocaine toxicity |
| **94** | Norfentanyl = 24 pg/mg | Gunshot wound of head |
| **95** | Fentanyl = 84 pg/mg  Norfentanyl = 10 pg/mg  4-ANPP = 11 pg/mg | Gunshot wound of back |
| **96** | Fentanyl = 2,162 pg/mg  Norfentanyl = 53 pg/mg  Acetyl fentanyl = 63 pg/mg  ß-hydroxy fentanyl = 79 pg/mg  Despropionyl para-fluorofentanyl = 26 pg/mg  4-ANPP = 273 pg/mg | Fentanyl, cocaine, ketamine toxicity |
| **97** | Fentanyl = 29 pg/mg | Fentanyl and methamphetamine toxicity |
| **98** | Fentanyl = 684 pg/mg  Norfentanyl = 9 pg/mg  4-ANPP = 410 pg/mg | Fentanyl and methamphetamine toxicity |
| **99** | Fentanyl = 13 pg/mg  4-ANPP < LOQ | Fentanyl toxicity |
| **100** | Fentanyl = 39 pg/mg  4-ANPP < LOQ | Gunshot wound of neck |
| **101** | Norfentanyl = 1,022 pg/mg | Hypertension and restrictive lung disease |
| **102** | Fentanyl = 468 pg/mg  Norfentanyl = 14 pg/mg  ß-hydroxy fentanyl = 13 pg/mg  4-ANPP = 140 pg/mg  Xylazine = 208 pg/mg | Methamphetamine, cocaine, fentanyl toxicity |
| **103** | Fentanyl = 7,504 pg/mg  Norfentanyl = 682 pg/mg  ß-hydroxy fentanyl = 410 pg/mg  Despropionyl para-fluorofentanyl = 1,385 pg/mg  4-ANPP = 534 pg/mg  Xylazine = 1,230 pg/mg | Methamphetamine, fentanyl, fluoro fentanyl toxicity |
| **104** | Fentanyl = 1,030 pg/mg  Norfentanyl = 10 pg/mg  Despropionyl para-fluorofentanyl < LOQ  4-ANPP = 249 pg/mg | Cocaine and fentanyl toxicity |
| **105** | Fentanyl = 140 pg/mg  4-ANPP = 12 pg/mg | Fentanyl and cocaine toxicity |
| **106** | Fentanyl = 2,735 pg/mg  Norfentanyl = 46 pg/mg  4-ANPP = 291 pg/mg | Fentanyl and methamphetamine toxicity |
| **107** | Norfentanyl = 15 pg/mg | Gunshot wound of head |
| **108** | Fentanyl = 4 pg/mg  4-ANPP < LOQ | Gunshot wound of neck and torso |
| **109** | Fentanyl = 317 pg/mg  Norfentanyl = 62 pg/mg  4-ANPP = 33 pg/mg | Multiple gunshot wounds |
| **110** | Fentanyl = 56 pg/mg  Norfentanyl = 18 pg/mg | Fentanyl toxicity |
| **111** | Fentanyl = 46 pg/mg  Norfentanyl = 12 pg/mg | Fentanyl toxicity |
| **112** | Fentanyl = 2,728 pg/mg  Norfentanyl = 856 pg/mg  ß-hydroxy fentanyl = 474 pg/mg  Despropionyl para-fluorofentanyl < LOQ  4-ANPP = 96 pg/mg  Xylazine = 1,826 pg/mg | Fentanyl toxicity |
| **113** | Fentanyl = 5 pg/mg | Fentanyl and cocaine toxicity |
| **114** | Fentanyl = 24 pg/mg  Norfentanyl = 15 pg/mg | Fentanyl, ethanol, cocaine toxicity |
| **115** | Fentanyl = 383 pg/mg  Norfentanyl = 20 pg/mg  4-ANPP = 25 pg/mg | Fentanyl and methamphetamine toxicity |
| **116** | Fentanyl = 4,866 pg/mg  Norfentanyl = 1,316 pg/mg  Acetyl fentanyl = 73 pg/mg  ß-hydroxy fentanyl = 315 pg/mg  Despropionyl para-fluorofentanyl = 18 pg/mg  4-ANPP = 822 pg/mg  Xylazine = 3,990 pg/mg | Cardiac tamponade |
| **117** | Fentanyl = 91 pg/mg  4-ANPP < LOQ | Ethanol, cocaine, fentanyl toxicity |
| **118** | Fentanyl = 34 pg/mg  4-ANPP < LOQ | Fentanyl and methamphetamine toxicity |
| **119** | Fentanyl = 12 pg/mg | Ruptured saccular aneurysm |
| **120** | Fentanyl = 35 pg/mg  Norfentanyl = 11 pg/mg | Methamphetamine toxicity |
| **121** | Fentanyl = 186 pg/mg  Norfentanyl = 46 pg/mg  4-ANPP = 13 pg/mg | Undetermined |
| **122** | Fentanyl = 186 pg/mg  Norfentanyl = 46 pg/mg | Fentanyl and methamphetamine toxicity |
| **123** | Fentanyl = 2,675 pg/mg  Norfentanyl = 151 pg/mg  ß-hydroxy fentanyl = 99 pg/mg  4-ANPP = 110 pg/mg  Xylazine = 149 pg/mg | Blunt force injuries |
| **124** | Fentanyl = 17 pg/mg  Norfentanyl = 14 pg/mg | Fentanyl and hydrocodone toxicity |
| **125** | Fentanyl = 51 pg/mg  Norfentanyl = 18 pg/mg | Arteriosclerotic cardiovascular disease |
| **126** | Fentanyl = 47 pg/mg  4-ANPP < LOQ | Gunshot wound of torso |
| **127** | Protonitazene = 24 pg/mg | Protonitazene toxicity |
| **128** | Fentanyl > 10,000 pg/mg  Norfentanyl = 335 pg/mg  Acetyl fentanyl = 431 pg/mg  ß-hydroxy fentanyl = 179 pg/mg  Despropionyl para-fluorofentanyl = 428 pg/mg  4-ANPP = 8,401 pg/mg  Xylazine = 3,514 pg/mg | Fentanyl toxicity |
| **129** | Fentanyl = 200 pg/mg  Norfentanyl = 13.5 pg/mg  4-ANPP = 90 pg/mg  Xylazine = < LOQ | Fentanyl, xylazine, methamphetamine toxicity |
| **130** | Fentanyl = 102 pg/mg  Norfentanyl = 9 pg/mg  4-ANPP < LOQ  Xylazine = 1,178 pg/mg | Fentanyl, heroin, xylazine toxicity |

*Supplementary data:. Hair NPFs concentrations and causes of death in the investigated population.*
